# Intrauterine transmission of SARS-CoV-2 to and prenatal ultrasound abnormal findings in the fetus of a pregnant woman with mild COVID-19

**DOI:** 10.1186/s12884-023-06053-y

**Published:** 2023-10-11

**Authors:** Meixiang Zhang, Liqiong Hou, Liangyu Guo, Qichang Zhou, Hougang Zhou, Na Sang, Ting Tan, Yan Xie, Yongjun Wang, Xiaoliang Huang, Jing Liu, Chunwang Li, Beilei Huang, Yulin Peng, Yifan Kong, Yingchun Luo

**Affiliations:** 1 Department of Ultrasound, The Maternal and Child Health Care Hospital of Hunan Province, Changsha, Hunan China; 2 Department of Ultrasound Diagnosis, The Second Xiangya Hospital of Central South University, Changsha, Hunan China; 3 Clinical Laboratory, The Maternal and Child Health Care Hospital of Hunan Province, Changsha, Hunan China; 4 Department of Pathology, The Maternal and Child Health Care Hospital of Hunan Province, Changsha, Hunan China; 5 Department of Blood Transfusion, The Second Xiangya Hospital of Central South University, Changsha, Hunan China; 6 Department of genetics, The Maternal and Child Health Care Hospital of Hunan Province, Changsha, Hunan China; 7 Department of Radiology, Hunan Children’s Hospital, Changsha, Hunan China

**Keywords:** COVID-19, Intrauterine transmission, Fetal abnormal ultrasound findings

## Abstract

**Background:**

Whether intrauterine transmission of COVID-19 occurs remains uncertain, and it remains unclear whether the disease affects fetuses. We present a case of intrauterine transmission of SARS-CoV-2 infection and the prenatal ultrasonographic findings of the fetus in a pregnant woman with mild COVID-19.

**Case presentation:**

A 30-year-old woman was admitted to our hospital for ultrasound examination in January 2023 at 26^+ 3^ weeks’ gestation. Twenty-one days prior, her COVID-19 nucleic acid test was positive, and she had mild symptoms, including fever (38.3 °C), headache, chills, ankle pain and cough. After receiving symptomatic treatment, she fully recovered. Prenatal ultrasound revealed that the placenta was diffusely distributed with punctate echogenic foci, hepatomegaly, and the volume of bilateral lungs decreased significantly, with enhanced echo. In addition, we found that the surface of the fetal brain demonstrated widened gyri with a flattened surface. The prenatal MRI confirmed these fetal abnormalities. Amniotic fluid was tested for SARS-CoV-2, and the sample tested was positive for the virus. After careful consideration, the pregnant woman decided to terminate the pregnancy.

**Conclusion:**

The intrauterine transmission of COVID-19 is certain. Moreover, the intrauterine transmission of COVID-19 may cause abnormalities in various organs of the fetus.

## Background

Since December 2019, COVID-19 has spread rapidly across the world and has been transmitted mainly by respiratory droplets, direct contact with fomites, and close person-to-person contact. However, to date, whether intrauterine transmission of COVID-19 occurs remains uncertain, and it remains unclear whether the disease affects fetuses. Some studies have found severe acute respiratory syndrome coronavirus 2 (SARS-CoV-2) in placental tissue, amniotic fluid and cord blood. However, there seems to be no report of abnormal prenatal ultrasound and MRI in pregnant women with COVID-19. We present a case of intrauterine transmission of SARS-CoV-2 infection and the prenatal ultrasonographic findings of the fetus in a pregnant woman with mild COVID-19. This case illustrates that the vertical transmission of COVID-19 does occur and that regular prenatal ultrasonography follow-up is essential to assess the potential risk to the fetuses of pregnant women diagnosed with COVID-19.

## Case presentation

**Fig. 1:**
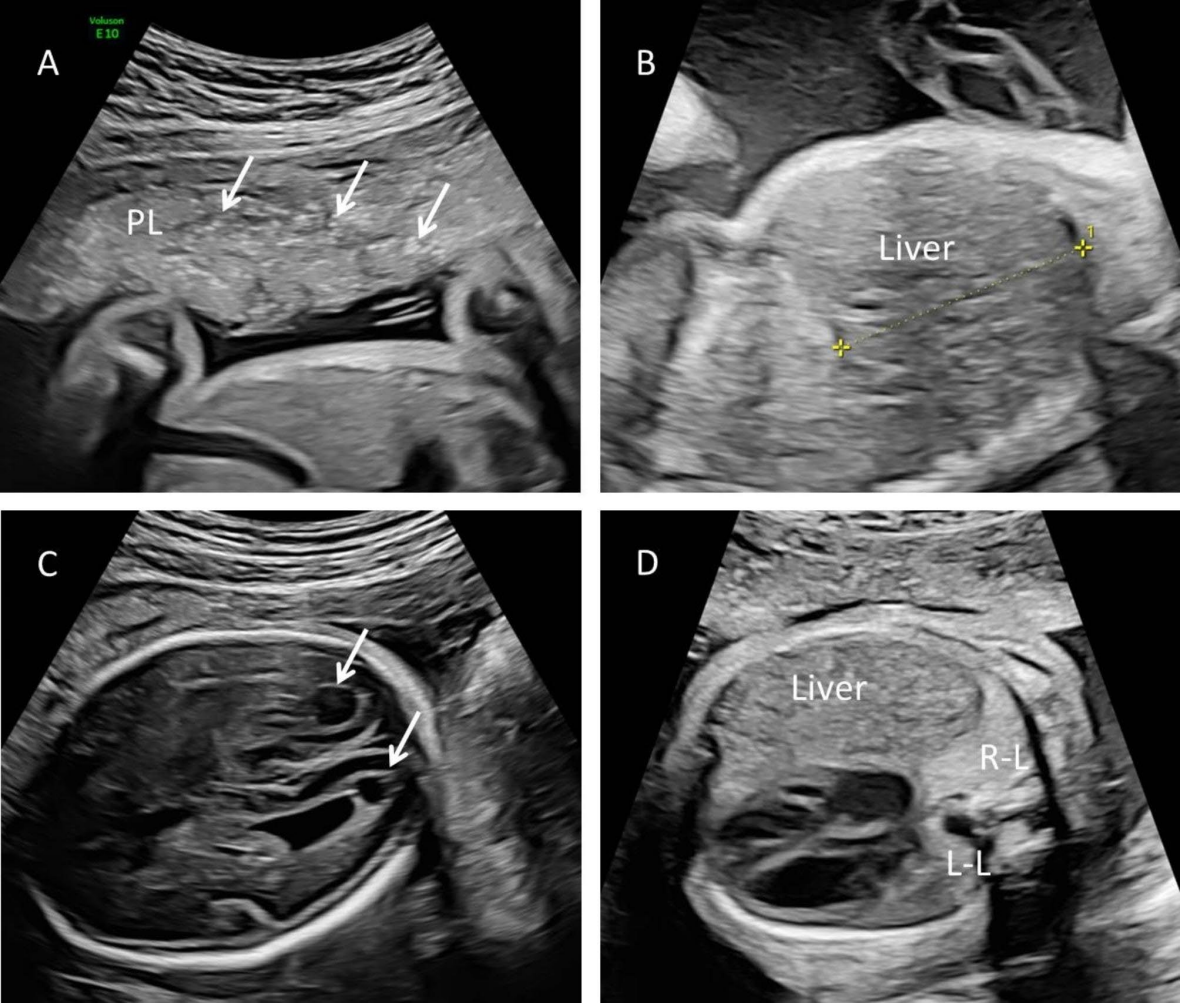
Prenatal ultrasound performed at 26^+ 3^ weeks’ gestation. (**A**) The placenta was diffusely distributed with punctate echogenic foci (arrows). (**B**) Sagittal view showed that the liver was significantly enlarged. (**C**) A subependymal cyst was seen in the posterior horn of the left and right lateral ventricles. (**D**) The transverse section of the chest showed that the bilateral lungs were significantly reduced and the echo was enhanced. PL, placenta. R-L, right lung. L-L, left lung

**Fig. 2:**
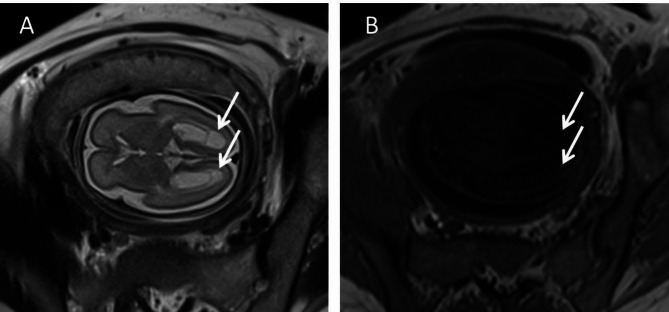
Prenatal MRI of fetal brain. (**A**) and (**B**) T2 and T1 weighted imaging, respectively. Images show subependymal cysts in the posterior horn of bilateral lateral ventricles (arrows)

**Fig. 3:**
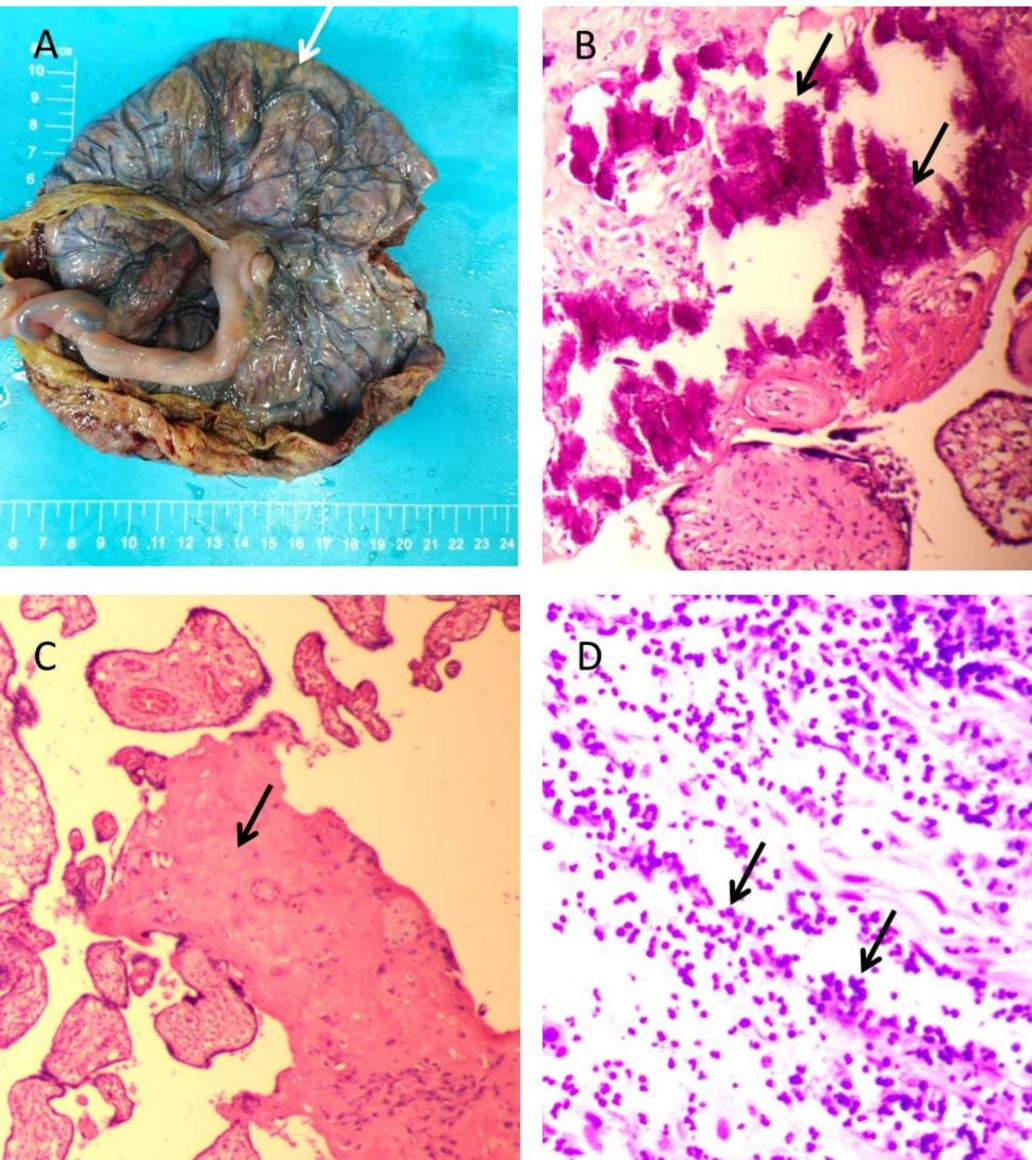
Gross and microscopic examination of the placenta. (**A**) The gross view of the placenta showed visible calcification (arrow). (**B**) Microscopic lesions of calcification (arrows) (HE stain, magnification 20 × 10). (**C**) Microscopic lesions of perivillous fibrin deposition, as a homogeneous red-stained area (arrow). (**D**) Microscopic lesions of acute intervillositis, mainly made of neutrophils (arrows)

**Fig. 4:**
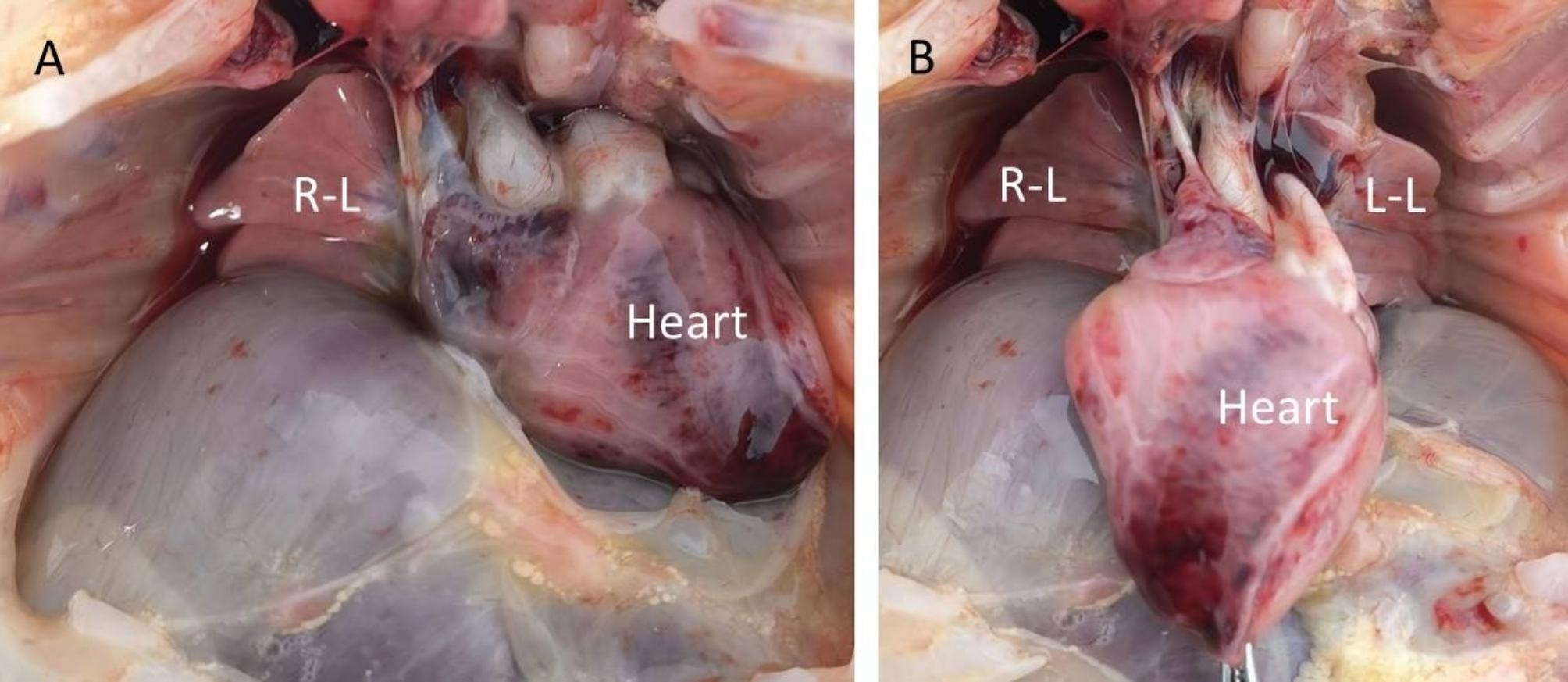
Gross anatomy of bilateral lungs. (**A**) On the general view of the bilateral lungs after opening the chest, it can be seen directly that the right lung was significantly reduced and the left lung was completely covered fetus by the heart. (**B**) After pulling the heart to the middle of the chest, the left lung could be completely displayed, and the bilateral lungs, especially the left lung, were obviously atrophic

**Fig. 5:**
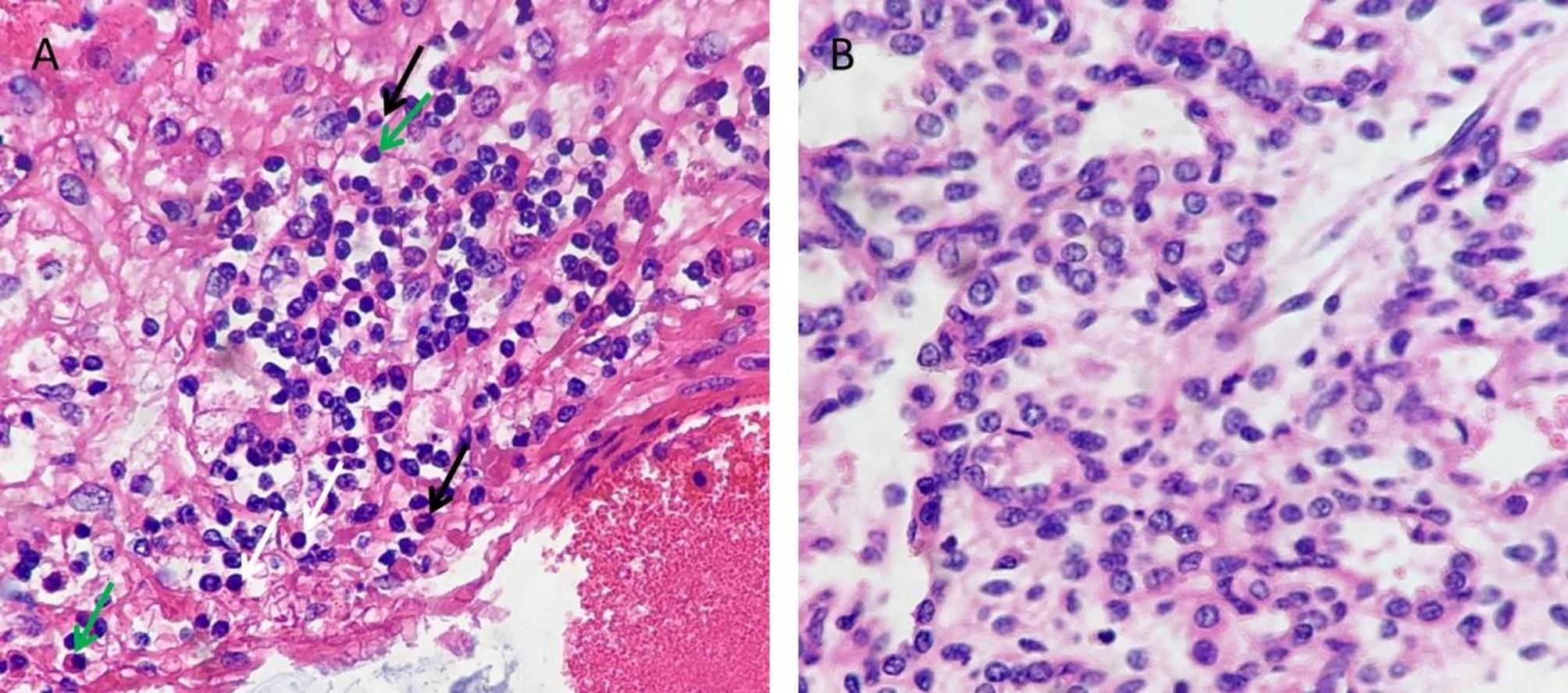
The microscopic examination of the fetal liver and lung. (**A**) There were a large number of inflammatory cells infiltration in the portal area, including neutrophils (black arrows), lymphocytes (white arrows) and plasma cells (green arrows) (HE stain, magnification 40 × 10). (**B**) Pathological examination of the lung showed that interlobular septa were poorly formed and some alveoli were poorly developed

A 30-year-old woman, gravida 1, para 0, was admitted to our hospital for ultrasound examination in January 2023 at 26^+ 3^ weeks’ gestation. She completed the third dose of the COVID-19 vaccine, produced in China, in December 2021. Twenty-one days prior, her COVID-19 nucleic acid test was positive, and she had mild symptoms, including fever (38.3 °C), headache, chills, ankle pain and cough. After receiving symptomatic treatment, she fully recovered. Prenatal ultrasound revealed that the placenta was diffusely distributed with punctate echogenic foci (Fig. 1a), and the volume of bilateral lungs decreased significantly, with enhanced echo. The sizes of the left and right lungs of the fetus were 1.6 cm×0.8 cm×1.0 and 2.2 cm×1.0 cm×1.3 cm, respectively (Fig. 1d). In addition, we found that the surface of the fetal brain demonstrated widened gyri with a flattened surface (Fig. 1c). A subependymal cyst was seen in the posterior horn of the left and right lateral ventricles, with sizes of 0.6 cm×0.6 cm×0.5 and 1.0 cm×0.8 cm×0.7 cm, respectively. The fetal liver was enlarged, with a vertical diameter of approximately 4.2 cm (Fig. 1b). The patient underwent the prenatal magnetic resonance imaging (MRI) examination in the external hospital, and the MRI confirmed these fetal abnormalities (Fig. 2 showed bilaterally subependymal cyst). After careful consideration, the pregnant woman decided to terminate the pregnancy. Amniotic fluid was tested for SARS-CoV-2 at 27^+ 2^ weeks’ gestation, and the sample tested was positive for the virus. TORCH was negative for IgM in the venous blood of pregnant women, while herpes simplex virus (HSV)-IgG, rubella virus (RV) IgG, and cytomegalovirus (CMV)-IgG were slightly higher than the upper limit value, and toxoplasma gondii (TOX)-IgG was negative. However, we performed the CMV and TOX DNA quantification in amniotic fluid using CMV and TOX nucleic acid detection kits, respectively, and the results were negative. After induced labor, the fetus was delivered through the vagina at 27^+ 3^ weeks’ gestation. After the delivery of the fetus, we immediately collected the venous blood of the pregnant woman and the fetal heart blood for quantitative detection of SARS-CoV-2 IgG/IgM, and the results showed that the IgG of both was significantly higher than the normal value. Their values were 11.925 S/CO and 8.609 S/CO respectively (reference value < 1.000 S/CO). Placental histological examination revealed perivillous fibrin deposition and multifocal calcification in the villi (Fig. 3). Other findings included acute chorioamnionitis (stage 1). With the written consent of the pregnant woman, we conducted an autopsy on the induced fetus. Gross anatomy showed significant reduction in bilateral lungs, especially in the left lung (Fig. 4). Results in fetal liver (Fig. 5a), there were a large number of inflammatory cells infiltration in the portal area, including neutrophils, lymphocytes and plasma cells. Pathological examination of the lung showed that interlobular septa were poorly formed and some alveoli were poorly developed, which indicated pulmonary hypoplasia (Fig. 5b).

## Discussion and conclusion

We report a proven case of intrauterine transmission of SARS-CoV-2 and prenatal abnormal findings in the fetus of a pregnant woman with mild COVID-19 in the second trimester. The consequences of COVID-19 infection in pregnant women and the potential risks of vertical transmission have become a major issue, and related research is also controversial. A review published in 2020 pointed out that there was minimal evidence of vertical transmission of COVID-19, although SARS-CoV-2 PCR was positive in one neonate 36 h after birth and another had evidence of SARS CoV-2 IgM and IgG 2 h after birth [1]. Despite a few studies have found SARS-CoV-2 in placental tissue, amniotic fluid and cord blood [2–5], more studies have reported that no SARS-CoV-2 was found in amniotic fluid, umbilical cord blood or neonatal throat swab samples, which indicates that intrauterine transmission is unlikely to occur [6–9]. In our case, SARS-CoV-2 was detected in amniotic fluid. According to the classification system of SARS-CoV-2 infection in pregnant women, fetuses and neonates, a congenital infection is considered proven if SARS-CoV-2 is detected in the amniotic fluid collected prior to the rupture of membranes or in blood drawn early in life; therefore, our case fully qualifies as intrauterine transmitted SARS-CoV-2 infection. In addition, our research showed that fetus born to mother with COVID-19 by induced labour can have detectable SARS-CoV-2 IgG. The presence of IgG may be due to the passive transfer of this immunoglobulin from the mother to the fetus across the placenta, or it may be caused by infection of the fetus. However, SARS-CoV-2 virus was present in the amniotic fluid of the pregnant woman who had tested positive for COVID-19, and the pathological examination showed placental inflammation. Therefore, we infer that SARS-CoV-2 IgG is more likely produced in response to the fetal infection. Furthermore, most of the patients in published studies developed symptoms and were diagnosed with COVID-19 in the third trimester, and few studies assessed risks in the first and second trimesters. In this case, we have demonstrated that intrauterine transmission of SARS-CoV-2 infection is possible in the second trimester.

To the best of our knowledge, this is the first report of prenatal ultrasound abnormalities of fetuses through vertical transmission of COVID-19. In our case, prenatal ultrasound examination showed that the placenta was diffusely distributed with punctate echogenic foci, which is extremely rare in the second trimester. This may be related to the multifocal calcification and perivillous fibrin deposition in the pathological examination of the placenta. Many studies have shown that viral infections during pregnancy lead to numerous intrauterine and fetal effects, especially during the first and second trimesters. For instance, rubella virus and cytomegalovirus infection causes congenital malformations in the first and second trimesters [10, 11]. Previous studies confirmed that SARS-CoV-2 infects target host cells by binding with cell membrane angiotensin converting enzyme II (ACE2), which is a membrane-bound aminopeptidase that is found in most organs, including the placenta, heart, lung and brain [12, 13]. However, previous studies did not mention secondary changes in the fetuses of pregnant women with COVID-19 in utero. In our case, prenatal ultrasound revealed fetal hepatomegaly, pulmonary dysplasia and intracranial abnormities, which were confirmed by MRI and autopsy. Whether the fetal liver, lung and brain are the target organ of intrauterine transmission of SARS-CoV-2 remains to be confirmed by further research. It also remains unclear whether these prenatal abnormalities are direct effects of SARS-CoV-2 or are immune-mediated secondary changes.

In conclusion, given these findings, we think that the intrauterine transmission of COVID-19 is certain. Moreover, the intrauterine transmission of COVID-19 may cause abnormalities in various organs of the fetus. We suggest that a prospective, multi-centre cohort study be conducted on a larger population sample in the future. Given the potential risks of COVID-19 to the fetus, we, especially obstetricians, must pay sufficient attention to this issue, and pregnant women should take extra precautions to protect themselves and their unborn children. For pregnant women diagnosed with COVID-19, regular prenatal ultrasonography follow-up is essential to assess the potential risk to the fetus.

## Data Availability

All data generated or analyzed during this study are included in this published article.

## Abbreviations

MRI: magnetic resonance imaging
ACE2: angiotensin converting enzyme II
TORCH: Toxoplasma gondii (TOX), rubella virus (RV), cytomegalovirus (CMV), herpes simplex virus (HSV)
